# Editorial: Exploring cutaneous drug-related and drug-associated adverse events: from clinical insight to therapeutic management

**DOI:** 10.3389/fmed.2026.1876851

**Published:** 2026-05-21

**Authors:** Maurizio Romagnuolo, Sergio Crovella, Haissam Abou-Saleh, Chiara Moltrasio

**Affiliations:** 1 Dermatology Unit, Fondazione IRCCS Ca' Granda Ospedale Maggiore Policlinico, Milan, Italy; 2 Department of Medical Biotechnologies, University of Siena, Siena, Italy; 3 Department of Biomedical Sciences, Health Cluster, Qatar University, Doha, Qatar; 4 Department of Environmental and Prevention Science, University of Ferrara, Ferrara, Italy; 5 Medical and Health Sciences Office, QU-Health, Qatar University, Doha, Qatar

**Keywords:** drug-related ADRs and adverse reactions, immune checkpoint inhibitor (ICI), JAK inhibitor, paradoxical reaction, ruxolitinib cream, skin drug reaction, topical steroid

Skin reactions account for the majority of drug-related adverse events and represent a frequent occurrence in routinary dermatological practice. During recent years novel and targeted therapies have revolutionized many medical fields, but they have also introduced unique cutaneous toxicities [e.g., paradoxical reactions to biologics and immune-related adverse events of immune checkpoint inhibitors (ICIs)]. These side effects present significant diagnostic and therapeutic challenges for clinicians trying to manage adverse events while treating the primary disease. Conversely, they also offer valuable opportunities to further understand the underlying pathophysiology of various skin conditions. The goal of this Research Topic was to gather and summarize the latest evidence on drug-induced and drug-associated dermatological manifestations for both classic medications and novel targeted therapies.

The majority of the articles (6/21) in our Research Topic focused on ICIs related cutaneous adverse events (AEs): Duan et al. described the case of a 72-year-old man who developed psoriasis 6 weeks after receiving the Programmed Death receptor-1 (PD-1) inhibitor camrelizumab for esophageal cancer, successfully resolved with drug discontinuation and topical anti-psoriatic therapy. Sun et al. documented an additional case of camrelizumab-associated psoriasiform skin reaction and reviewed the literature on camrelizumab cutaneous AEs, highlighting reactive cutaneous capillary endothelial proliferation (RCCEP) as a prevalent and specific cutaneous adverse drug reaction (in up to 60% of the patients). Buyukahisha et al. provided an educational case report describing the occurrence of cutaneous lichen planus in a 43-year-old patient treated with the programmed death-ligand 1 (PD-L1) pembrolizumab for a triple negative breast cancer, while Didona et al. successfully used the anti-interleukin (IL)-4/IL-13 inhibitor dupilumab in a case series of 4 patients with ICIs-induced bullous pemphigoid, also identifying BP180 midportion as a specific autoantigenic target in this specific subset of patients.

Wang described a case of toxic epidermal necrolysis (TEN) following PD-1 inhibitor serlupimab administration for a small cells lung cancer (SCLC), focusing on the integrated multidisciplinary management with Western and Chinese nursing care and providing an interesting and educational perspective on the traditional Chinese medicine principles and practical approaches to skin diseases. Regarding Stevens-Johnson syndrome (SJS)/TEN spectrum—a rare but life-threating severe cutaneous adverse reaction—Pu et al. retrospectively analyzed 21 cases which followed ICIs administration, providing a detailed description of baseline characteristics, culprit dugs, clinical manifestation, systemic complication, laboratory and histopathological findings and treatment outcomes. The authors showed a median latency of 28 days from drug initiation to clinical onset, suggesting that a prompt diagnosis, allowing for immediate ICIs discontinuation and systemic corticosteroids initiation—often in combination with intravenous immunoglobulins (IVIG)—could significantly reduce the mortality in SJS/TEN patients.

An additional contribution to the SJS/TEN field came from Nikitina et al. The authors investigated the predictive value of individual components of severity-of-illness score for toxic epidermal necrolysis (SCORTEN) in a real-world setting: they retrospectively analyzed 150 SJS/TEN patients medical records and performed a univariate analysis between in-hospital death (overall mortality 18.7%) and each SCORTEN parameter, showing that only tachycardia, malignancy and high serum urea remained independent predictors of mortality following multivariate regression analysis. These findings suggest that some SCORTEN parameters may be recalibrated to improve risk stratification in the current therapeutic context.

The second most cited class of drug in our Research Topic articles was Janus Kinase inhibitors (JAKi), which represent a breakthrough in the treatment of inflammatory skin diseases. Both potential cutaneous side effects of JAKi and their use as a “rescue therapy” in drug-related skin AEs have been documented in our Research Topic.

Tang et al. conducted a retrospective disproportionality analysis using the US Food and Drug Administration Adverse Events Reporting System (FAERS) database on the association between oral JAK-1 inhibitors and infection risk in atopic dermatitis. Their findings confirmed the established association with herpetic infections and reactivations (e.g., herpes simplex and herpes zoster), pneumonia and influenza virus, but also suggested potential unlabelled association with sepsis and appendicitis, which requires further confirmation in real-life setting and independent studies.

Regarding the infection risk of JAKi, we reported the case of a 53-year-old man with vitiligo who developed tinea faciei at the application site of ruxolitinib 1.5% cream, which eventually led to the permanent discontinuation of therapy (Romagnuolo et al.). Topical ruxolitinib represents a novel and effective topical JAK1/2 inhibitor approved for non-segmental vitiligo with a generally favorable safety profile: clinical trials only reported mild-to-moderate AEs including site-application acne, nasopharyngitis and site-application pruritus, although real-world data on infection risk are still lacking.

JAKi site-application acne is also discussed in the timely and up-to-date review on acneiform drug eruption conducted by Ulrich et al.: in this review the authors provide a comprehensive overview of the different class of drugs implicated in acneiform rash, focusing on the clinical and histopathological features of this entity, while also detailing differential diagnosis, current pathophysiology and proposed therapeutic strategies.

On the other hand, systemic JAKi have proved effective in treating paradoxical reaction triggered by biologics. Tang and Liu presented the case of a 33-year-old man with ankylosing spondylitis (AS) who developed a pustular eruption mainly involving palms and soles while taking the IL-17 inhibitor secukinumab; this eruption was successfully resolved by discontinuing secukinumab and introducing the JAK-1 inhibitor upadacitinib. Similarly, Fang and Pan successfully treated a 25-year-old woman with AS who developed a severe eczematous reaction while on secukinumab by switching her to upadacitinib. These cases highlight the utility of JAKi for treating both biologic-induced skin reactions and comorbid conditions.

A further contribution on biologics-adverse reaction has been reported by, Zhao et al., who performed a disproportionality analysis for evaluating the safety of the anti-IL-17A ixekizumab basing on the post-market report of AEs retrieved from the FAERS and VigiAccess databases. This study confirmed known AEs (e.g., fungal infections, injection site reactions, upper respiratory tract infections and inflammatory bowel diseases) but also potential association with other AEs such as cellulitis, herpes zoster, ear infection and diverticulitis, which warrant further real-world investigation.

Xi et al. documented the occurrence of bullous pemphigoid in a 78-year-old woman affected by psoriasis following treatment with the anti-IL-23 guselkumab, while also performing a comparative single cell transcriptomic analysis on tissue samples of both diseases, in order to reveal distinctive cellular landscape and disrupted pathways associated with this reaction.

In addition to novel and emerging treatment-related AEs, our Research Topic highlights 3 articles dedicated to topical steroids, which underscores their widespread use and valid role in current dermatological practice.

Chen et al. reported a rare case of iatrogenic Cushing syndrome, likely induced by the abuse of topical clobetasol propionate over a 5-year for psoriasis treatment, suggesting that physician should remain vigilant regarding topical steroid therapies prescribed to patients, as well as monitoring the use of over-the-counter medications both for patients' safety and for a thorough anamnesis during clinical practice.

On the similar subject of topical steroid abuse or “addiction,” Myles and Ratley published a perspective on topical steroid withdrawal (TSW) syndrome, covering its history and varying definitions, as well as the academic scepticisms and controversies surrounding it. Furthermore, they outline current knowledge gaps in TWS and propose criteria to assist both patients and clinicians in recognizing this debated condition.

Finally, a retrospective study on 55 male lichen sclerosus et atrophicus (LSA) of the genitalia has been proposed by Pessina et al. This study evaluated the effects of topical steroid vs. circumcision on quality of life (QoL) and post-operative recurrence risk. The authors reported that topical steroids were not significantly associated with QoL improvement measured by dermatological life quality index (DLQI) compared to circumcision and that the median steroid-free survival after circumcision was 19 months. Nevertheless, they concluded that topical steroids remain a mainstay treatment across the various stages of LSA, emphasizing the need for a tailored treatment in LSA patients.

Other contributions covering topics beyond those mentioned above reflect the broad spectrum of cutaneous drug reactions, especially in oncological patients.

Dudík et al. reported the case of a 63-year-old woman with metastatic cervical cancer who experienced a large-volume paclitaxel extravasation during a peripheral infusion, treated with warm dry compresses and local hyaluronidase; despite the benefit, neuropathic complications could not have been prevented due to the high neurotoxicity of paclitaxel. This case underscores the importance of administering vesicant chemotherapy via a central venous catheter rather than peripheral line, to reduce extravasation risk.

Zinc supplementation has been proposed as a supportive measure to mitigate enfortumab vedotin (EV) symmetrical distributed drug-related intertriginous and flexural exanthema (SDRIFE)-like in urothelial cancer patients by Nishizawa et al.: the authors observed that SDRIFE-like rash occurred in 6 patients with lower serum zinc levels compared to those without the drug reaction, and 3 out of 6 patients improved within days despite poor response to topical corticosteroids.

Another contribution by Zhang et al. described the occurrence of a neutrophilic dermatosis compatible with Sweet Syndrome in a patient with myelofibrosis treated with Granulocyte-colony stimulating factor (G-CSF). Notably, the lesions also appeared at surgical incision and drainage sites suggesting a Koebner's phenomenon; furthermore, SS presentation preceded the patient's final diagnosis of acute myeloid leukemia by several months.

Lastly Zhou et al. basing on a literature review and a two-round expert consensus meeting constructed a management and prevention programme for target therapy-induced hand-foot skin reaction. This programme should offer practical nursing care guidelines with the aim to deliver personalized and symptoms-driven management strategies for patients experiencing this condition.

In conclusion, this Research Topic highlights how the shift toward targeted therapies and immunotherapies has redefined the landscape of cutaneous adverse events, introducing complex diagnostic challenges, but also opportunities for pathophysiological inquiry on drug-related skin adverse events. The evidence presented underscores the complexity of ICI-induced reactions and the dual role of JAK inhibitors as both potential triggers of adverse reaction and effective rescue therapies for biologic-induced paradoxical eruptions. Furthermore, the diverse cutaneous toxicities in oncological patients emphasize the need for a multidisciplinary and highly specialized management in this setting. Ultimately, integrating active pharmacovigilance with real-world clinical data is essential to optimizing therapeutic continuity and improving safety outcomes for patients receiving novel and targeted pharmacological agents.

